# Prognostic impact of *fibroblast growth factor receptor 2* gene amplification in patients receiving fluoropyrimidine and platinum chemotherapy for metastatic and locally advanced unresectable gastric cancers

**DOI:** 10.18632/oncotarget.12953

**Published:** 2016-10-27

**Authors:** Seyoung Seo, Seong Joon Park, Min-Hee Ryu, Sook Ryun Park, Baek-Yeol Ryoo, Young Soo Park, Young-Soon Na, Chae-Won Lee, Ju-Kyung Lee, Yoon-Koo Kang

**Affiliations:** ^1^ Department of Oncology, Asan Medical Center, University of Ulsan College of Medicine, Seoul, Korea; ^2^ Department of Pathology, Asan Medical Center, University of Ulsan College of Medicine, Seoul, Korea; ^3^ Asan Institute for Life Science, Asan Medical Center, University of Ulsan College of Medicine, Seoul, Korea

**Keywords:** FGFR2, amplification, advanced gastric cancer, prognosis, quantitative real-time polymerase chain reaction

## Abstract

Although *Fibroblast growth factor receptor (FGFR) 2* gene amplification and its prognostic significance have been reported in resectable gastric cancers, information on these features remains limited in the metastatic setting. The presence of *FGFR2* amplification was assessed in formalin-fixed, paraffin-embedded tissues using a quantitative PCR-based gene copy number assay with advanced gastric cancer cohorts. A total of 327 patients with tumor portion of ≥70% were analyzed for clinical features. Among these patients, 260 who received first-line fluoropyrimidine and platinum chemotherapy were analyzed for survival.

Sixteen of 327 patients (4.9%) exhibited *FGFR2* amplification. The amplification group showed associations with age <65 years, Borrmann type 4 disease, poor performance status, poorly differentiated histology, extra-abdominal lymph node metastases, and bone metastases. The median overall survival (OS) and progression-free survival (PFS) were found to be 12.7 and 5.8 months, respectively. In univariate analysis, PFS did not differ between amplification and no amplification groups (hazard ratio [HR]=1.34, 95% confidence interval [CI]: 0.78-2.31, *p*=0.290), although the OS was significantly shorter in the amplification group (HR=1.92, 95% CI: 1.13-3.26, *p*=0.015). However, multivariate analysis indicated that *FGFR2* amplification was not an independent prognostic factor for OS (HR=1.42, 95% CI: 0.77-2.61, *p*=0.261).

Although *FGFR2* amplification is associated with poorer OS, it does not appear to be an independent prognostic predictor in patients with advanced gastric cancer treated with palliative fluoropyrimidine and platinum chemotherapy.

## INTRODUCTION

Although the prognosis of advanced gastric cancer has improved after introduction of cytotoxic chemotherapy [1], gastric cancer remains one of the leading causes of cancer-related death worldwide [2, 3]. Various chemotherapeutic agents have been investigated for the management of metastatic gastric cancer, including infusional 5-fluorouracil, CDDP, anthracyclines, taxanes, oral fluoropyrimidines, and oxaliplatin [4–8]. At present, a standard chemotherapy protocol for gastric cancer has not been established; however, the combination of CDDP and infusional 5-fluorouracil, with or without epirubicin, is most commonly prescribed, and is considered as a reference treatment by most regulatory agencies when evaluating newer treatments. Furthermore, several phase III trials have demonstrated that oral fluoropyrimidines (such as S-1 and capecitabine) can replace infusional 5-fluorouracil in the treatment of gastric cancer [6–9].

Given the disappointing clinical outcomes of malignant gastric cancer, targeted treatments are being actively investigated. The combination of trastuzumab with chemotherapy in HER2-positive advanced gastric cancer patients as first-line therapy and the addition of ramucirumab to taxane in non-selective advanced gastric cancer patients as second-line therapy exhibited modest survival benefits [10, 11], whereas other targeted agents, including bevacizumab, everolimus, and cetuximab, did not show overall survival gain without the use of biomarker enrichment strategies [12–14]. Despite these efforts to improve survival in gastric cancer, most patients with advanced gastric cancer usually have a median overall survival (OS) of < 12 months. Thus, a considerable amount of research is required to discover novel treatment targets for patients with advanced gastric cancer.

The regulation of the fibroblast growth factor (FGF) signaling pathway is important for normal growth control, and the genetic alteration of the FGF receptor (FGFR) reportedly enhances downstream signaling and is related to tumorigenesis [15, 16]. In particular, an increase in the *FGFR2* copy number was reported in cases of breast cancer [17, 18] and poorly differentiated gastric cancer [19, 20]. Furthermore, a few studies examined the clinicopathologic features of *FGFR2-*amplified gastric cancer and showed that *FGFR2* amplification was associated with poorer prognosis [21–23]. Accordingly, *FGFR2* amplification was considered as a reasonable treatment target and predictive biomarker for small molecule tyrosine kinase inhibitors or antibodies to FGFR2, including dovitinib, BGJ398, Ki23057, AZD4547, and GP369 [24–28]. However, previous studies on gastric cancer were conducted in patients with localized resectable gastric cancer. Hence, these findings cannot be directly applied to patients with recurrent or unresectable gastric cancer who are indicated for palliative chemotherapy.

Fluorescence *in situ* hybridization (FISH) is considered as the standard method for detecting gene amplification. However, due to the high cost and long procedure duration of FISH testing, real-time quantitative polymerase chain reaction (qPCR)-based gene copy number assay was suggested as a possible alternative to detect *FGFR2* amplification [21, 29]. In our previous study, we showed that the *FGFR2/*CEP10 ratio, determined *via* FISH, were very well correlated with the results of the qPCR-based gene copy number assay, with a cut-off value of 8 for *FGFR2* amplification [29].

In the present study, we aimed to investigate the association of *FGFR2* amplification with the clinicopathologic features and prognostic significance in patients with unresectable gastric cancer treated with fluoropyrimidine and platinum (FP) as first-line chemotherapy. Moreover, we assessed the *FGFR2* amplification status by using qPCR, a sensitive but less expensive method.

## RESULTS

### Patient characteristics

**Figure 1:**
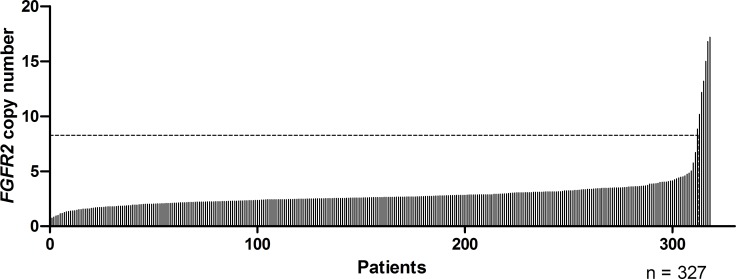
*FGFR2* copy numbers determined with a quantitative PCR-based assay in metastatic or locally advanced gastric cancer *FGFR2* copy number of ≥ 8 was observed in 16 cases and 9 data points are outside the axis limits on this graph.

The formalin-fixed paraffin-embedded (FFPE) samples of a total of 327 patients had a tumor portion of > 70%, and were adequate for analyzing the relationship between *FGFR2* amplification and clinicopathologic factors. The patients had a median age of 58 years (range, 23-85 years); moreover, 68.8% of patients had initially metastatic disease, whereas the remaining presented with recurring and locally advanced unresectable disease. At the time of diagnosis, 288 (88.1%) patients had an Eastern Cooperative Oncology Group (ECOG) performance status of 0-1 (Table 1). The median copy numbers on *FGFR2* qPCR was 2.64 (range, 0.73-504.04) and the frequency of *FGFR2* amplification was 4.9% (*n* = 16) (Figure 1).

**Table 1:** Baseline characteristics of the study patients (*n* = 327)

		*n*	(%)	Median (range)
*FGFR2* qPCR value	gene copy number ≥8	16	4.9	2.64 (0.73–504.04)
Gender	Male	226	69.1	
Age	≥65 years	93	35.9	58 (23–85)
ECOG PS	0 or 1	228	88.1	
Borrmann type	I	17	5.2	
	II	71	21.7	
	III	178	54.4	
	IV	50	15.3	
	Not available	11	3.4	
Histology	WD/MD	117	35.8	
	PD/SRC/mucinous	204	62.4	
	Others	6	1.8	
HER2/neu^a^	Positive	19	5.8	
	Negative	103	31.5	
	Not tested	205	62.8	
No gastrectomy		211	64.5	
Disease status	Initially metastatic	215	68.8	
	Recurred	99	30.3	
	Locally advanced	13	0.9	
Metastatic organ	Peritoneum	157	48.0	
	Liver	95	29.1	
	Lung	20	6.1	
	Intraabdominal distant LN	154	47.1	
	Extra-abdominal distant LN	31	9.5	
	Bone	27	8.3	
Hemoglobin^b, c^	≤lower normal limit	223	68.3	11.7 (6.7–17.4)
White blood cell^c^	≥10000/mm^3^	47	14.4	6850 (2200–48700)
Platelet^c^	≤150×10^3^/mm^3^	38	11.9	264 (14–646) × 10^3^
Albumin^d^	<3.3 g/dL	104	31.8	3.6 (1.7–5.3)
Alkaline phosphatase^c^	>120 IU/L	71	21.7	79.5 (29–1294)
Total bilirubin ^c^	>1.2 mg/dL	29	8.9	0.6 (0.2–6)
Risk groups ^c,e^	Good (0–1)	154	47.1	
	Moderate (2–3)	112	34.3	
	Poor (≥4)	52	15.9	

Abbreviations: qPCR, quantitative polymerase chain reaction; *ECOG PS*, Eastern Cooperative Oncology Group Performance Status; *WD*, well differentiated; *MD*, moderately differentiated; *PD*, poorly differentiated; *SRC*, signet ring cell carcinoma; *LN*, lymph node

^a^HER2/neu-positive was defined as a positive score of ≥2 on immunohistochemistry and/or positive results from silver in situ hybridization

^b^Hemoglobin ≤12 g/dL for women and ≤13 g/dL for men.

^c^Initial complete blood count, alkaline phosphatase level, bilirubin level, and scores of the Asan Medical Center prognostic model were not available for 9 patients (2.8%).

^d^Albumin levels were not available in 11 patients (3.4%)

^e^According to the Asan Medical Center prognostic model

### Association between *FGFR2* amplification and the clinicopathologic features

The clinical characteristics were compared between the patients with and without *FGFR2* amplification. The amplification group showed an association with age < 65 years (93.6% *vs*. 70.4%, *p* = 0.047), ECOG performance status ≥ 2 (31.3% *vs*. 10.9, *p* = 0.031), Borrmann type 4 disease (43.7% *vs*. 13.7%, *p* = 0.013), poorly differentiated pathology including signet ring cell and mucinous carcinoma (87.5% *vs*. 62.3%, *p* = 0.041), extra-abdominal lymph node metastases (31.3% *vs*. 6.4%, *p* = 0.011), and bone metastases (31.3% *vs* 7.1%, *p* = 0.006). After stratifying the patients according to risk by using our previously described prognostic model for metastatic or recurrent gastric cancer [30], we found that the amplification group was more closely related to the poor prognostic group (43.8% *vs*. 14.9%, *p* = 0.004) (Table 2).

**Table 2:** Relationship between *c* amplification and the clinicopathologic features (*n* = 327)

		*FGFR2* gCN of <8	*FGFR2* gCN of ≥8	*p*
		(*n* = 311, 95.1%)	(*n* = 16, 4.9%)	
Age	Median (range)	58 (23–85)	50.5 (32–66)	
	<65 years	219 (70.4)	15 (93.8)	0.047
	≥65 years	92 (29.6)	1 (6.3)	
Gender	Male	216 (69.5)	10 (62.5)	0.584
	Female	95 (30.5)	6 (37.5)	
ECOG PS	0–1	277 (89.1)	11 (68.8)	0.031
	2–4	34 (10.9)	5 (31.3)	
Bormann type	I/II/III	257 (85.7)	9 (56.3)	0.006
	IV	43 (14.3)	7 (43.8)	
Histology	WD/MD	115 (37.7)	2 (12.5)	0.041
	PD/SRC/mucinous	190 (62.3)	14 (87.5)	
Peritoneal metastasis	No	164 (52.7)	6 (37.5)	0.234
	Yes	147 (47.3)	10 (62.5)	
Liver metastasis	No	218 (70.1)	14 (87.5)	0.166
	Yes	93 (29.9)	2 (12.5)	
Lung metastasis	No	292 (93.9)	15 (93.8)	1.0
	Yes	19 (6.1)	1 (6.3)	
Intraabdominal distant LN metastasis	No	163 (52.4)	10 (62.5)	0.43
	Yes	148 (47.6)	6 (37.5)	
Extra-abdominal distant LN metastasis	No	285 (91.6)	11 (68.8)	0.011
	Yes	26 (8.4)	5 (31.3)	
Bone metastasis	No	289 (92.9)	11 (68.8)	0.006
	Yes	22 (7.1)	5 (31.3)	
Hemoglobin^a,b^	>LNL	89 (29.5)	6 (37.5)	0.576
	≤LNL	213 (70.5)	10 (62.5)	
White blood cell ^b^	<10000/mm^3^	257 (85.1)	14 (87.5)	1.0
	≥10000/mm^3^	45 (14.9)	2 (12.5)	
Platelet ^b^	>150×10^3^/mm^3^	268 (88.7)	12 (75.0)	0.11
	≤150×10^3^/mm^3^	34 (11.3)	4 (25.0)	
Albumin^c^	>3.3 g/dL	202 (67.1)	10 (66.7)	1.0
	≤3.3 g/dL	99 (32.9)	5 (33.3)	
Alkaline phosphatase ^b^	≤120 IU/L	235 (77.8)	12 (75.0)	1.0
	>120 IU/L	67 (22.2)	4 (25.0)	
Total bilirubin ^b^	≤1.2 mg/dL	274 (90.7)	15 (93.8)	1.0
	>1.2 mg/dL	28 (9.3)	1 (6.3)	
Risk groups ^b,d^	Good	147 (48.7)	7 (43.8)	0.004
	Moderate	110 (36.4)	2 (12.5)	
	Poor	45 (14.9)	7 (43.8)	

Abbreviations: *gCN*, gene copy numbers; *ECOG PS*, Eastern Cooperative Oncology Group Performance Status; *WD*, well differentiated; *MD*, moderately differentiated; *PD*, poorly differentiated; *SRC*, signet ring cell carcinoma; *LN*, lymph node; *LNL*, lower normal limit

^a^Hemoglobin ≤12 g/dL for women and ≤13 g/dL for men.

^b^Initial complete blood count, alkaline phosphatase level, bilirubin level, and scores of the Asan Medical Center prognostic model were not available for 9 patients (2.8%).

^c^Albumin levels were not available in 11 patients (3.4%)

^d^According to the Asan Medical Center prognostic model

### Association between *FGFR2* amplification and survival outcome

A total of 260 patients treated with the FP regimen were included in the survival analysis; among these patients, 172 presented with measurable lesions. An objective response was observed in 81 of 172 patients (47.7%), and the overall response rate did not significantly differ between the amplification and no amplification groups (55.6% *vs*. 43.9%, *p* = 1.000). Overall, 88.8% of the patients had died at the time of analysis. Over a median follow-up of 28.2 months (range, 8.2-68.5 months), the median OS and progression free survival (PFS) durations were 12.7 months (95% confidence interval [CI]: 11.0-14.5) and 5.8 months (95% CI: 4.8-6.8), respectively.

**Figure 2:**
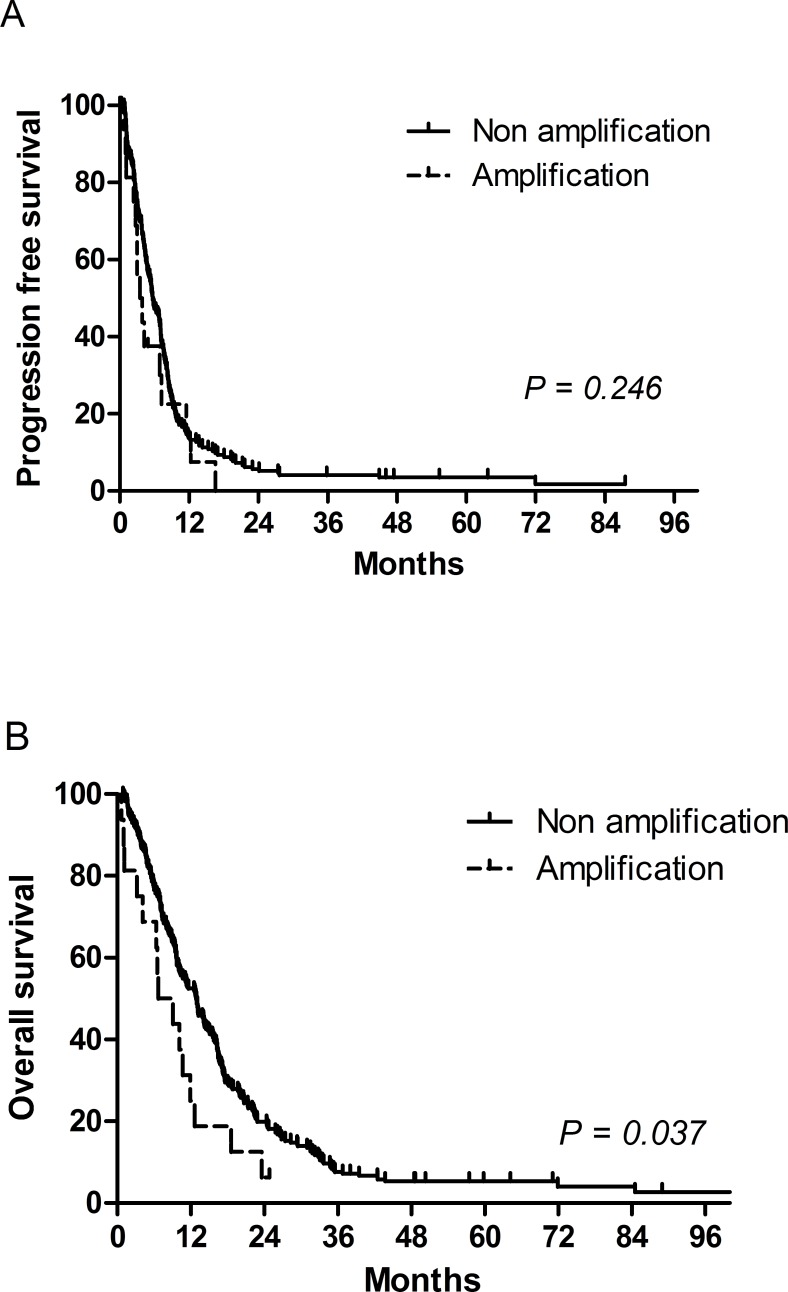
Progression-free survival and overall survival according to *FGFR2* amplification **A**. progression free survival was not significantly different between *FGFR2* amplification group and no amplification group. **B**. *FGFR2* amplification was associated with shorter overall survival.

Univariate analysis did not indicate PFS difference between the amplification and no amplification groups (hazard ratio [HR] = 1.34, 95% CI: 0.78-2.31, *p* = 0.290), although the OS duration was significantly shorter in the amplification group (HR = 1.92, 95% CI: 1.13-3.26, *p* = 0.015) (Figure 2). Patients who did not undergo gastrectomy and those with Borrmann type 4 disease, bone metastasis, low albumin levels, or elevated alkaline phosphatase (ALP) levels exhibited poorer PFS. The following factors were significantly associated with a shorter OS: ECOG performance status ≥ 2, no gastrectomy, Borrmann type 4 disease, bone metastasis, lung metastasis, elevated ALP levels, and low albumin levels (Table 3). However, when the other significant prognostic factors were included, multivariate analysis showed that *FGFR2* amplification was not an independent prognostic factor of OS (HR = 1.42, 95% CI: 0.77-2.61, *p* = 0.261). In fact, multivariate analysis indicated that Bormann type IV disease, lung metastasis, and elevated ALP levels were not associated with poor OS, although no gastrectomy, poor ECOG performance status, bone metastasis, and low albumin levels remained significant prognostic factors (Table 4). In addition, when the patients were stratified by risk based on the recommendations of our previous report [30], *FGFR2* amplification was not found to be significantly associated with OS (HR = 1.61, 95% CI: 0.94-2.77, *p* = 0.083) or PFS (HR = 1.26, 95% CI: 0.72-2.19, *p* = 0.418), although risk stratification did show a prognostic significance for OS (moderate risk group: HR = 1.35, 95% CI, 1.01-1.81, *p* = 0.042; poor risk group: HR = 3.07, 95% CI, 2.12-4.43, *p* < 0.001) (Table 4).

**Table 3:** Univariate analysis of progression-free and overall survival (*n* = 260)

		Progression-free survival			Overall survival		
		HR	95% CI	*p*	HR	95% CI	*p*
Gender	Female	1.02	0.76–1.37	0.907	0.99	0.74–1.33	0.952
Age	≥65 years	0.88	0.65–1.21	0.434	1.17	0.89–1.56	0.267
ECOG PS	2–3	1.52	1.00–2.32	0.052	2.64	1.82–3.82	<0.001
Bormann type	IV	1.95	1.36–2.81	<0.001	1.76	1.25–2.48	0.001
PD/SRC/ mucinous histology		1.25	0.93–1.69	0.139	1.12	0.85–1.48	0.411
No gastrectomy		1.64	1.21–2.21	0.001	1.99	1.47–2.69	<0.001
Peritoneal metastasis		0.98	0.74–1.28	0.853	1.17	0.90–1.53	0.233
Liver metastasis		1.12	0.83–1.51	0.475	1.09	0.82–1.45	0.551
Lung metastasis		1.85	1.00–3.40	0.048	1.83	1.02–3.28	0.042
Intraabdominal distant LN		0.99	0.76–1.30	0.938	0.89	0.69–1.16	0.382
Extra-abdominal distant LN		1.03	0.65–1.66	0.891	1.32	0.84–2.07	0.227
Bone metastasis		2.46	1.55–3.90	<0.001	3.53	2.28–5.47	<0.001
Hemoglobin^a^	≤LNL	0.79	0.59–1.06	0.116	0.99	0.75–1.31	0.934
WBC	≥10000/mm^3^	1.01	0.67–1.51	0.971	1.07	0.72–1.59	0.747
Platelet	≤150×10^3^/mm^3^	0.98	0.64–1.49	0.906	1.15	0.77–1.73	0.499
Albumin	≤3.3 g/dL	1.53	1.15–2.03	0.003	2.22	1.69–2.91	<0.001
ALP	>120 IU/L	1.64	1.18–2.28	0.003	1.88	1.38–2.55	<0.001
Total bilirubin	>1.2 mg/dL	1.29	0.82–2.03	0.267	1.31	0.86–1.98	0.206
Risk groups^b^	Good	1			1		
	Moderate	1.12	0.83–1.50	0.476	1.33	0.99–1.78	0.055
	Poor	1.94	1.31–2.87	0.001	3.19	2.22–4.58	<0.001
*FGFR2* qPCR	gCN ≥8	1.34	0.78–2.31	0.290	1.92	1.13–3.26	0.015

Abbreviations: *HR,* hazard ratio; *CI,* confidence interval; *ECOG PS*, Eastern Cooperative Oncology Group Performance Status; *PD*, poorly differentiated; *SRC*, signet ring cell carcinoma; *LN*, lymph node; *LNL*, lower normal limit; *WBC*, white blood cells; *ALP*, alkaline phosphatase; *qPCR*, quantitative polymerase chain reaction; *gCN*, gene copy number

^a^Hemoglobin ≤12 g/dL for women and ≤13 g/dL for men.

^b^According to the Asan Medical Center prognostic model

**Table 4:** Multivariate Cox proportional hazard models to confirm the prognostic significance of *FGFR2* amplification with other clinical factors (*n* = 260)

		Hazard ratio	95% confidence interval	*P*
No gastrectomy		1.47	1.05–2.39	0.025
Albumin <3.3 g/dL		1.62	1.20–2.19	0.002
ECOG PS ≥2		1.68	1.13–2.50	0.011
Borrmann type IV		1.40	0.98–1.99	0.062
Bone metastasis		2.49	1.58–3.91	<0.001
Lung metastasis		1.87	0.98–3.57	0.059
*FGFR2* qPCR gCN ≥8		1.61	0.94–2.77	0.083
Risk groups^a^	Good	1		
	Moderate	1.35	1.01–1.81	0.042
	Poor	3.07	2.13–4.43	<0.001

Abbreviations: *ECOG PS*, Eastern Cooperative Oncology Group Performance Status; *qPCR*, quantitative polymerase chain reaction; *gCN*, gene copy numbers

^a^According to the Asan Medical Center prognostic model

## DISCUSSION

We determined the presence of *FGFR2* amplification by using the qPCR-based gene copy number assay, and found that *FGFR2* amplification was present in 4.9% of the patients with metastatic or locally advanced unresectable gastric cancer. *FGFR2* amplification was also associated with proven poor prognostic factors of gastric cancer, including poor performance status and bone metastases. Although *FGFR2* amplification was significantly associated with a shorter OS, it was not found to be an independent poor prognostic factor in patients with metastatic and locally advanced unresectable gastric cancer, and could not predict the chemotherapy response. To our knowledge, our present study is the largest retrospective analysis to date of the clinical and prognostic implications of *FGFR2* amplification in metastatic or locally advanced unresectable gastric cancer. Notably, the prognostic impact of *FGFR2* amplification observed in the present study did not markedly differ from that in previous studies on advanced gastric cancer patients who received palliative chemotherapy [31, 32]. However, current results are distinct from those of studies on resectable gastric cancer, which reported a relationship between *FGFR2* amplification and poor prognosis [21–23].

Previous studies have reported that the FGFR2 protein was overexpressed by immunohistrochemistry (IHC) in 30-40% of gastric cancer patients undergoing curative resection [33, 34], although the incidence of *FGFR2* gene amplification was only 4-10 % in cases of resectable gastric cancer [21–23, 26, 35] and 4.4-11.5% in cases of unresectable gastric cancer [31, 32]. Our frequency of *FGFR2* amplification was consistent with that described in previous reports. The discordance in the reported incidences between protein expression and gene amplification could be explained by the heterogeneous amplification of *FGFR2* in tissues or the varied antibodies and standard protocols used for IHC [29].

In present study, *FGFR2* amplification was found to be associated with several clinicopathologic parameters, including younger age, poor ECOG performance status, extra-abdominal lymph node metastasis, bone metastasis, poorly differentiated histology, and Bormann type IV disease. These factors might reflect high tumor burden or aggressive biology, and have been suggested as poor prognostic factors in patients with advanced gastric cancer [30, 36]. Consistent with current findings, previous studies have indicated an association between high-grade histology and *FGFR2* amplification in resectable gastric cancer [22, 23, 37]. However, the relationship between age and *FGFR2* amplification is controversial, as prior studies have reported both a significant association between *FGFR2* amplification and older age [21] and no such relationship [22, 23]. Furthermore, we found that *FGFR2* amplification was related to a higher risk group by using our prognostic model that was developed by combining multiple clinicopathologic features of advanced gastric cancer [30]. These findings suggest that *FGFR2* amplification is associated with negative prognostic factors in advanced gastric cancer, and could act as a confounding factor when we analyze its effect on survival.

Because some patients in the present study were diagnosed before 2012, we were unable to obtain information regarding HER2 expression or amplification in these cases. After conducting a cross-correlation analysis to assess the association between *FGFR2* and *HER2* amplification, while allowing for the missing data, we found that these 2 factors were not related (data not shown). In fact, none of our patients presented with both *FGFR2* and *HER2* amplification, consistent with previous reports that have described the mutual exclusivity of these conditions [22, 32, 35, 37, 38].

Su et al. reported that *FGFR2* amplification was more frequently observed in gastric cancer patients with a higher N stage and poorly differentiated histology [22]; these factors are considered to contribute to the development of recurrence and poor prognosis in *FGFR2*-amplified resectable gastric cancer. Although it can be reasonably assumed that *FGFR2* amplification is a negative prognostic indicator in patients with resectable gastric cancer [21–23], these results cannot be applied to metastatic or locally advanced unresectable gastric cancer, as noted in the present and recently published studies [31, 32]. By using FISH testing, Shoji et al. showed that *FGFR2-*amplified gastric cancer patients in the palliative setting tended to have a shorter survival period. Even though the enrolled patients were treated with a heterogeneous treatment regimen, including trastuzumab or triplet regimens, the authors found a relationship between *FGFR2* amplification and poor OS, which was significant on univariate analysis but was not significant on multivariate analysis [31]. More recently, Matsusaka et al. also reported no significant correlation between survival outcomes and *FGFR2* amplification when using an arbitrarily determined cut-off value (copy number of 5) on qPCR [32]. Our current finding is consistent with those of previous studies, but we validated the reasonable cut-off value of a copy number of 8 on qPCR reported in our previous study to predict amplification *via* FISH [29]; hence, this study could suggest a more solid conclusion. In addition, our present study included the largest number of patients who received homogenous treatment, and could hence reliably indicate the survival outcome.

Although *FGFR2* amplification is expected to be a new therapeutic target for advanced gastric cancer [25–28], a recent randomized phase II study comparing the efficacy of AZD4547 *versus* paclitaxel for advanced gastric cancer with *FGFR2* amplification or polysomy did not show any significant benefits in the AZD4547 arm [39]. Notably, the authors observed marked intra-tumoral heterogeneity of *FGFR2* amplification, which could potentially explain the failure of AZD4547 treatment. Hence, *FGFR2* amplification is a questionable predictive marker for the response to FGFR2 inhibitor alone in metastatic or unresectable gastric cancer. Another possible hypothesis states that FGFR2 inhibitor monotherapy itself was not effective for advanced gastric cancer due to the presence of other escape mechanisms. Moreover, the FGFR2 inhibitor Ki23057 showed a synergistic effect with the chemotherapeutic agents in an *in vitro* test [40]. In addition, patients with FGFR2 protein expression determined by IHC, exhibited a better response (85.7%) after combination treatment with pazopanib, capecitabine, and oxaliplatin in a phase II trial, in comparison with patients without FGFR2 protein expression (59.5%) in advanced gastric cancer [41]. As the accompanying *FGFR2* amplification did not affect the chemotherapy response in advanced gastric cancer in present and previous studies [31, 32], it could be hypothesized that the FGFR2 inhibitor would be more effective when combined with cytotoxic agents. Given the discordance between *FGFR2* gene amplification and protein expression, this explanation is needed to validate by qPCR or FISH method in future trials.

Although FISH is considered the standard method for the diagnosis of gene amplification, the qPCR-based gene copy number assay has been found to be a reasonable alternative for detecting *FGFR2* amplification [21, 29]. For qPCR of the *FGFR2* gene in tissues with a tumor portion of < 70%, microdissection will be needed. However, the qPCR-based method is less expensive and has comparable sensitivity, and can hence be adopted for broad practical use. We believe our current results were also reliable and applicable in practice.

In conclusion, *FGFR2* amplification is not an independent prognostic predictor in patients with metastatic or locally advanced gastric cancer treated with palliative FP. Further validation is warranted to obtain a better clinical understanding of *FGFR2* amplification in patients with gastric cancer treated with palliative chemotherapy.

## MATERIALS AND METHODS

### Patient samples and clinical data

Between June 2006 and December 2014, we screened 1367 patients who received palliative chemotherapy for metastatic or locally advanced unresectable gastric cancer and were registered in a single tertiary center gastric cancer registry. After a histological review, a total of 327 patients who had sufficient tissue specimens for qPCR and specimens comprised of ≥ 70% of tumor portion form pretreatment biopsied or surgically obtained FFPE tissues were selected for the analysis of the clinicopathologic features of *FGFR2-*amplified gastric cancer. Among these patients, 260 who were treated with first-line FP chemotherapy were analyzed for the prognostic impact of *FGFR2* amplification. The medical records of all these patients were reviewed. This study adhered to the guidelines established by the declaration of Helsinki, and was approved by our institutional review board.

### Isolation of genomic DNA and real-time qPCR-based determination of the gene copy number of *FGFR2*

Genomic DNA extraction, DNA concentration measurement, and real-time qPCR for determining the gene copy number of *FGFR2* were conducted in a similar manner as in our previous study [29]. Genomic DNA was extracted from biopsy specimens or surgical FFPE tissues using a QIAamp DNA FFPE Tissue kit or QIAamp DNA Mini kit (Qiagen, Hilden, Germany). The DNA concentration was measured using the NanoDrop 2000 spectrophotometer (Thermo Scientific, Waltham, MA). To determine the gene copy number of *FGFR2*, pre-designed TaqMan Copy Number Assays were used (Applied Biosystems). For real-time PCR, we prepared a total volume of 10 μL of master mixture, which contained 10 ng of genomic DNA, 5 μL of TaqMan genotyping master mix, and each primer. The primer IDs were HS05182482_cn (intron 14 and 15) and Hs05114211_cn (intron 12). The telomerase reverse transcriptase (TERT) gene and human genomic DNA (Takara) were used as internal references for the copy number and normal control, respectively. The thermal cycling conditions were as follows: 10 min at 95°C, followed by 40 cycles of 15 s at 95°C and 60 s at 60°C. The results were analyzed using the ABI PRISM 7900HT Sequence Detection System (Applied Biosystems).

### Statistical analysis

To analyze the relationship between *FGFR2* amplification and the clinical features and survival outcomes of gastric cancer, the patients were classified as having *FGFR2* amplification based on the presence of an *FGFR2* qPCR gene copy number of ≥ 8, according to our previous study [29]. PFS was defined as the duration between the start of FP chemotherapy and tumor progression or death by any cause. Moreover, OS was estimated from the date of the initial first-line FP session until death by any cause. Data were censored if the patients were free of progression or alive at the final follow-up. Categorical variables were evaluated using the chi-square test or Fisher’s exact test, as appropriate. The Kaplan-Meier method was used to estimate PFS and OS. Survival curves were compared using the log-rank test according to *FGFR2* amplification. By multivariate analysis, the Cox proportional hazard model was used, and we included potent prognostic factors: ECOG Performance Status ≥ 2, no gastrectomy, peritoneal metastasis, bone metastasis, lung metastasis, ALP > 120 IU/L, albumin < 3.3 g/dL, and total bilirubin > 1.2 mg/dL [30].

All statistical analyses were performed using the Statistical Package for the Social Sciences version 21 (IBM Co., Armonk, NY). All tests were two-sided with 5% defined as the level of significance.
